# A Diagnostic Procedure for Identifying Isotherm Models in Liquid Chromatography

**DOI:** 10.1021/acs.iecr.5c03704

**Published:** 2026-01-06

**Authors:** Konstantinos Katsoulas, Federico Galvanin, Luca Mazzei, Eva Sorensen

**Affiliations:** Department of Chemical Engineering, Sargent Centre for Process Systems Engineering, 4919 University College London, Torrington Place, London WC1E 7JE, United Kingdom

## Abstract

Liquid chromatography is a pivotal purification process widely used in pharmaceutical development and manufacturing. Efficient optimal design and control of the process rely heavily on mechanistic models such as the lumped pore diffusion model (POR) and the Equilibrium Dispersion Model (EDM), both popular choices owing to their simplicity and good accuracy for a wide range of applications. However, the choice of the functional form of the isotherm models, which describe the component adsorption equilibria, strongly affects the predictions of the chromatography model. While traditional isotherms perform well for simple compounds (e.g., small molecules), they often fall short for more complex separations (e.g., peptides), thus resulting in process-model mismatch, even following rigorous parameter estimation. As a remedy to this, recent advances have introduced hybrid models that integrate data-driven elements to improve the predictive accuracy, although at the cost of loss of process insight, low interpretability, and increased complexity. To address the process-model mismatch in chromatography, we have proposed a model diagnostic procedure, adapted from a diagnostic framework in kinetic models, based on a Lagrange multiplier test, to refine isotherm models that initially underfit. The procedure is demonstrated by three in-silico case studies, showing improved accuracy against experimental data without having to resort to black-box models, thus providing models that retain physical insight.

## Graphical abstract

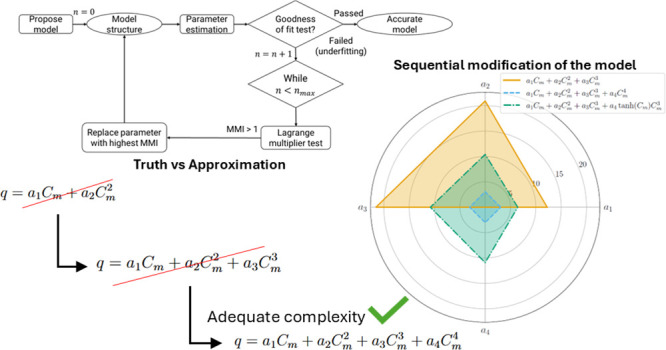

## Introduction

Liquid chromatography is one of the most established separation processes used in pharmaceutical drug development, employed for sample analysis in the early drug developments stages as well as for purification in the scale-up and manufacturing stages.^1–3^ Mathematical models can aid in the optimal design and operation of chromatographic separations, and thereby contribute to faster drugs-to-market by quickly and accurately developing reliable and sustainable separations by minimizing processing time, solvent usage, as well as maximizing product quality.^4,5^

Modelers rely on mechanistic models to capture the underlying phenomena of chromatographic separations.^6–10^ The existing literature has established a series of mechanistic, or first-principles, models that differ in accuracy and complexity. The most commonly employed models are the Equilibrium Dispersion Model (EDM) and lumped pore diffusion model (POR), which rely on a system of partial differential and algebraic equations.^11^ These equations encapsulate the separation mechanism and predict the chromatograms of the components under consideration. Separation of the components occurs due to the different affinities of the components in the mobile phase toward the stationary phase, and the affinity is dependent on the different adsorption–desorption mechanism of each component. Therefore, model-based development of the process requires equations that are reliable and representative in describing the underlying phenomena, e.g. adsorption and desorption. For instance, the EDM usually assumes that adsorption and desorption are instantaneous, and thus simply described by an isotherm model.^5^ The EDM also assumes that pore mass transfer is extremely fast and accounts for it in an apparent dispersion coefficient.^11^ On the other hand, the POR model, although it also relies on an isotherm model, does not assume extremely fast mass transfer, and thus it describes the mass transfer process via a differential equation; e.g., a linear driving force model.^12,13^ Overall, the adsorption and desorption, and pore mass transfer are the focal point of model-based chromatography method development.

For years, modelers have successfully used fundamental isotherms (e.g., Langmuir) to capture the adsorption equilibrium.^14–16^ Although fundamental isotherms are a good approach for simple molecules vis-à-vis accuracy, more complex molecules (e.g., amino-acids, small peptides, proteins, etc.) might require isotherms (and potentially mass transfer resistance models) that do not adhere to the classic model structures that are so well established in the literature. Modelers, in that case, must select less conventional isotherm model structures (e.g., Moreau, bi-Moreau, quadratic),^17–20^ although the isotherm model may still not be adequate to capture the underlying physics properly. Modelers have therefore shifted toward the use of hybrid models, which comprise physics-based as well as data-driven components. Recent literature in the field of chromatography has revealed that it is possible to replace the isotherm model or the mass transfer kinetics model (or both) of the POR or the EDM with a data-driven model. Specifically, Narayanan et al.^21,22^ developed an approach, working with the POR model, to replace established isotherms with Neural Networks (NNs) that could outperform conventional isotherm models in terms of interpolating and extrapolating simulated breakthrough curves in Protein A columns. According to Narayanan et al.,^21^ first-principle models can exhibit varying degrees of hybridization. Specifically, Narayanan et al. proposed that the knowledge-driven components of a POR model, namely the mass transfer kinetics, mass transfer coefficient, and adsorption isotherm, can be replaced by NNs, either individually or by lumping these components into a single data-driven equation for mass transfer kinetics. This approach can increase the degree of hybridization up to 100%. The authors remarked that hybrid models with an intermediate degree of hybridization tend to outperform purely data-driven models in terms of prediction accuracy and process interpretability. Although such models may sacrifice some of the mechanistic insight of purely knowledge-driven approaches, this trade-off is compensated by their better versatility and practical applicability. In the same context, Ding et al.^23^ proposed an approach to replace the isotherm model of EDM with a NN in the complex paradigm of salt-dependent Hydrophobic Interaction Chromatography (HIC) columns. However, replacing an isotherm or mass transfer kinetics model with a black box model loses its interpretability and thereby the insight into the fundamental principles of the separation process. In a system where the governing phenomena are described by a POR model, Santana et al.^24^ therefore replaced the mass transfer kinetics model with a NN, although they retrieved an equation through sparse regression to restore some of the lost interpretability. Applications of hybrid modeling in chromatography are not limited to replacing the isotherm or the mass transfer model with a surrogate (e.g., NNs), but also aim at integrating both the main mass balances and the binding models (mass transfer and adsorption) in a data-driven structure in order to make computationally efficient predictions.^25^

Hybrid modeling in chromatography, although functional, comes with certain limitations. Particularly, combining data-driven and first-principle models increases the overall model complexity, in terms of number of parameters. In addition, the data-driven components of the hybrid models can prove to be data hungry and pose the risk of overfitting. Moreover, regulated environments such as the pharmaceutical industry are often sceptical toward machine learning applications.^26^ Therefore, first-principle models should be favored over hybrid models unless the former cannot adequately capture the process behavior, that is, where there is process-model mismatch. In such cases, one could still rely on approximated models that are only valid within specific regions of the design space.^27^ Process-model mismatch is detected when the simulations underfit the experimental data and goodness-of-fit tests fail. Quaglio et al.^28^ proposed a procedure that diagnoses potential mismatch between kinetic models and experimental data and refines the models iteratively until a level of appropriate complexity is reached. If the proposed model underfits, the parameters of the model undergo a Lagrange multiplier test^29^ that can indicate which parameters can be substituted by state-dependent functions. The procedure is then repeated until the model simulations fit the experimental data satisfactorily. The methodology developed by Quaglio et al. is similar to the incremental identification techniques^30–32^ developed to decompose a large identification problem into smaller and simpler problems.

In this work, we have adapted the methodology proposed by Quaglio et al.^28^ to the needs of isotherm model identification for chromatographic separations. We use the EDM coupled with isotherm models that we modify iteratively according to what the Lagrange multiplier test indicates in each iteration until the model stops underfitting the experiments, resulting in a model that accurately describes the elution profiles in question and that can be used for optimal design and operation. The elution profiles in question exhibit a nonclassical adsorption behavior. Such profiles may belong to more complex molecules, such as amino-acids, small peptides, and proteins.^19,20,33^ Therefore, the use of conventional isotherms gives rise to process-model mismatch. Our approach can remedy this mismatch by adding additional degrees of complexity to the initial approximated model. Alternatively, in a pure hybrid approach, to model the adsorption isotherm, one uses an equation obtained via a data-driven method. This equation usually contains no physical meaning, while in our approach, we start from physically meaningful adsorption isotherms and, if necessary, we generalize them by replacing some of the parameters with functions of state variables to increase the model complexity to the level required to obtain accurate model predictions. Accurate and interpretable mathematical representations of the isotherm models lead to reliable and efficient model-based method development for purifications in both research and industrial applications.

This manuscript is structured as follows: First, an overview of the modeling components and the proposed diagnostic procedure is presented. The proposed diagnostic procedure is then implemented in three distinct case studies, and the results are discussed. Finally, some concluding remarks and future directions are offered.

## Methodology

This section outlines the methodology adopted in the proposed diagnostic procedure which is then subsequently used in the following case studies. The methodology considers theoretical foundations of the maximum likelihood estimation and the χ^2^ test, and expands further to the foundations of the Lagrange multiplier test. In addition, we present the chromatographic model used and explain notions of the isotherm models.

### Parameter Estimation

Chromatographic models usually employ a set of one-dimensional Partial Differential and Algebraic Equations (PDAEs). The set of equations involves an *N*_*s*_ dimensional vector of state variables (e.g., the mobile phase concentrations within the column), **x**(*t*, *z*), and three *N*_*s*_ dimensional vectors of the derivatives of **x**(*t*, *z*), namely the first-order derivatives with respect to time, **ẋ**(*t*, *z*), and space, **x**_*z*_(*t*, *z*), and the second-order derivative with respect to space, **x**_*zz*_(*t*, *z*). A process setup consists of an *N*_*u*_ dimensional vector of manipulated inputs (e.g., the inlet solvent composition), **u**(*t*), and an *N*_*w*_ vector of time-invariant inputs (e.g., the sample volume), **w**. The model also includes an *N*_θ_ dimensional vector of parameters, **θ**. The measured variables, **ŷ**, are grouped in an *N*_*y*_ dimensional vector that represents the model outputs that can also be directly measured through experiments (e.g., outlet mobile phase concentrations). Thus, we can write

$$ŷ=f\left[x\left(t,z\right),ẋ\left(t,z\right),x_{z}\left(t,z\right),x_{zz}\left(t,z\right),u\left(t\right),w,\theta \right]$$ 1

If we obtain a *Y* experimental data set, i.e. *Y* = [**y**_1_,..., **y**_*N*_], that consists of *N* number of experiments, we can estimate the parameters for the model under consideration. The experimental measurements are assumed to be associated with noise which can be described as Gaussian with *N*_*y*_ × *N*_*y*_ covariance Σ_*y*_. In this work, we estimate parameters through a maximum likelihood approach,^34^ that is by maximizing the unconstrained log-likelihood function:^28^

$$\mathrm{\theta ̂}=\arg\;\max_{\theta }\;\Phi \left(Y|\theta \right)$$ 2

The log-likelihood function reads:

$$\Phi \left(Y|\theta \right)=-\frac{N}{2}\left[N_{y}\ln\left(2\pi \right)+\ln\left(\det\left(\Sigma_{y}\right)\right)\right]-\frac{1}{2}\overset{N}{\underset{i=1}{\sum }}{\left[y_{i}-{ŷ}_{i}\left(\theta_{1},...,\theta_{N_{\theta }}\right)\right]}^{T}\Sigma_{y}^{-1}\left[y_{i}-{ŷ}_{i}\left(\theta_{i},...,\theta_{N_{\theta }}\right)\right]$$ 3

where **ŷ**_*i*_ is the model prediction for the *i*-th experiment. At the maximum likelihood estimates, **θ̂** = [θ̂_1_,...,θ̂_*N*_θ__], the gradient of the objective function (i.e., the log-likelihood) with respect to the parameters **θ** is zero:

∇Φ(Y|θ̂)=0 4

### The χ^2^ Test

When simulations are fitted to the experimental data set and the maximum likelihood estimates are obtained, the goodness-of-fit is assessed through a χ^2^ test.^35^ If the model under consideration is assumed to be exact, then the normalized square residuals, χ^2^, must be distributed as a χ^2^ distribution with a degree of freedom, DoF = *N* · *N*_*y*_ – *N*_θ_:^34^

χ2=∑i=1N[yi−y^i(θ̂)]TΣy−1[yi−y^i(θ̂)]∼χN·Ny−Nθ2 5

In this work, we only consider underfitting, that is, when a model is too simple to capture the experimental data accurately, and thus we implement a single-tailed χ^2^ test with 95% significance level. (We do not consider overfitting, since the models used are just complex enough to represent the system.) If the calculated χ^2^ lies above the 95% χ_*c*_^2^ critical value, the model is said to be underfitting, and thus fails the test. If the χ^2^ is below the limit of the 95% χ_*c*_^2^, then the model passes the test and is considered accurate.

### Diagnostic Procedure

When a model underfits, its complexity is not adequate enough to capture the underlying physics of the given process. Silvey^29^ proposed a tailored Lagrange multiplier test to determine whether the model parameters are independent of the state variables of the system under consideration. Essentially, underfitting can be explained if model parameters are in fact functions instead of constants, i.e. there are hidden dependencies on state variables. Quaglio et al.^28^ introduced a framework to remedy the underfit by increasing the model complexity iteratively by employing the Lagrange multiplier test. According to Figure 1, once parameter estimation has been performed and the χ^2^ test has been assessed, if the considered model fails the latter, one should proceed with the Lagrange multiplier test. The Lagrange multiplier statistic aids in the calculation of a heuristic metric, the Model Modification Index (MMI). The MMI captures the expected improvement of the goodness-of-fit of the model, with a “faulty” parameter (i.e., a parameter that is not independent of the system state) being replaced with a function of state variables. Faulty parameters yield MMI values above 1. The modeler must then replace the faulty parameter with a function dependent on state variables, and repeat the procedure until an appropriate model complexity has been reached. Generally, a single faulty parameter can cause high MMI values in itself and in other, otherwise unproblematic, model parameters. However, the MMI value of the “culprit” parameter will be larger than those of the “benign” parameters. Therefore, it is strongly recommended that modelers follow a one-by-one modification procedure starting from the parameter with the highest MMI value. To prevent an infinite loop of model modifications, the modeler can define a maximum number of iterations *n*_max_, which serves to terminate the procedure when a series of modifications fails to improve model accuracy. The value of *n*_max_ is an intuitive threshold that users can set and depends on the resources and time that can be spent on a particular problem of model modification.

**Figure 1:**
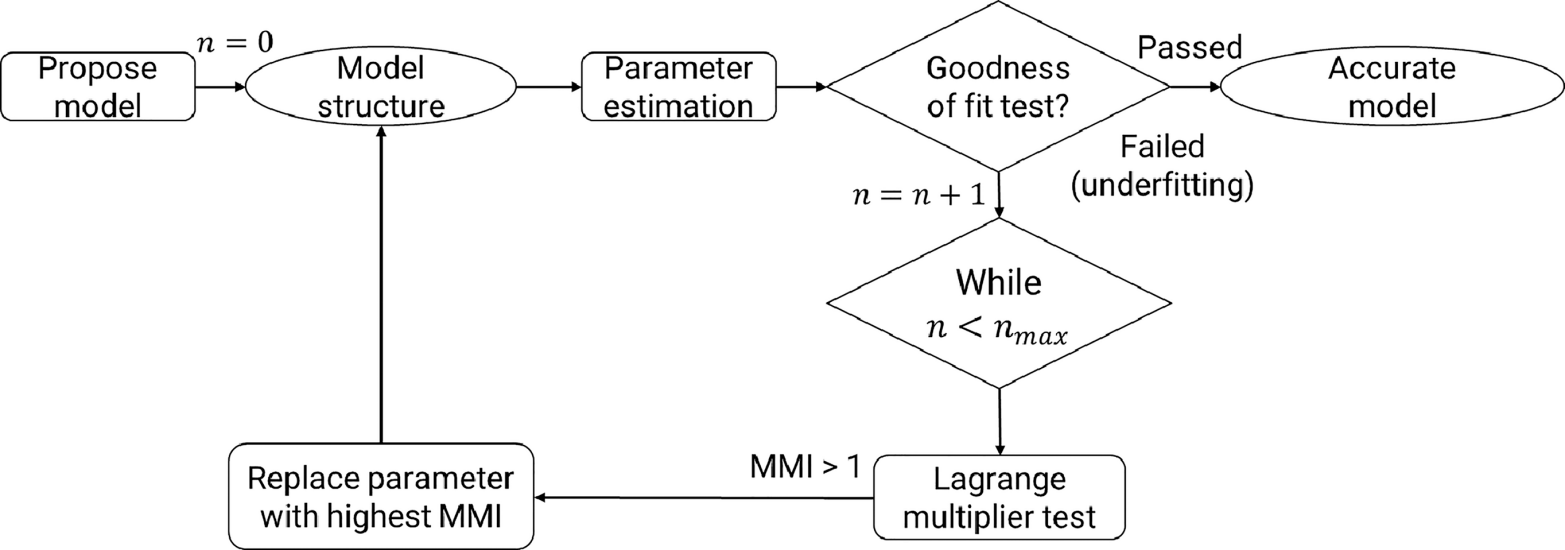
Proposed framework for model diagnosis and modification for underfitting models (*n*: number of iterations, MMI: Model Modification Index).

### Lagrange Multiplier Test and MMI

According to the proposed procedure, when a model fails to simulate an experimental data set accurately, the modeler needs to revisit the model equation and replace candidate parameters with a function of the state variables. But first, one needs to identify which parameters are not independent and are in reality functions of the state variables. In theory, the procedure can be applied for any model parameter that is considered for parameter estimation, but in our case we will consider isotherm models that are constitutive equations of chromatographic models. The Lagrange multiplier test^28^ assesses the following null hypothesis, in the instance where the parameter under consideration is the θ_*i*_ = θ_1_:

- *H*_0_: θ_1_ and θ_*j*_ ∀ *j*≠1 are all state-independent constants

with the alternative hypothesis stating:

- *H*_*a*_: θ_1_ is a state-dependent function and θ_*j*_∀ *j*≠1 are state-independent constants

Different experimental conditions impact the state variables of the system under consideration. Thus, the subvector of manipulated variables or time-invariant inputs that are considered for experiment design and parameter estimation, so the design vector, **φ**, impacts the state of the system. If θ_1_ is indeed a function *g* of the state variables, it is also a function of experimental conditions affecting the states, that is θ_1_ = *g*(**φ**), and θ_*j*_ ∀ *j*≠1 are fixed constants. To continue with the test, we need not assume any functional form for the *g* function. Let us also assume a theoretical *N* dimensional vector θ_*d*_ = [θ_1,1_,...,θ_1,*N*_] wherein the *i*-th element represents the value of θ_1_ at the different experimental conditions **φ**_*i*_, that is θ_1,*i*_ = *g*(**φ**_*i*_) ∀ *i* = 1,..., *N*. Under parametrization, **θ**_*d*_, the log-likelihood function, reads:

$$\Phi_{d}\left(Y|\theta_{d}\right)=-\frac{N}{2}\left[N_{y}\ln\left(2\pi \right)+\ln\left(\det\left(\Sigma_{y}\right)\right)\right]-\frac{1}{2}\overset{N}{\underset{i=1}{\sum }}{\left[y_{i}-{ŷ}_{i}\left(\theta_{1,i},{\mathrm{\theta ̂}}_{2}...,{\mathrm{\theta ̂}}_{N_{\theta }}\right)\right]}^{T}\Sigma_{y}^{-1}\left[y_{i}-{ŷ}_{i}\left(\theta_{1,i},{\mathrm{\theta ̂}}_{2}...,{\mathrm{\theta ̂}}_{N_{\theta }}\right)\right]$$ 6

Note that in the above eq (eq 6), all parameters θ_*j*_ ∀ *j*≠1 are fixed to their max-likelihood values, while parameter θ_1,*i*_ is assumed to vary across *N* experimental data sets, for the sake of performing the Lagrange multiplier test. To mathematically formalize the null hypothesis, we first need to introduce the *N* – 1-dimensional vector of functions:

$$s=\left[\theta_{1,1}-\theta_{1,2},...,\theta_{1,i}-\theta_{1,i+1},...,\theta_{1,N-1}-\theta_{1,N}\right]$$ 7

If the null hypothesis *H*_0_ is satisfied, the parameter θ_1_ is a constant, so that θ_1,*i*_ is equal to θ_1,*i*+1_ for any value of *i*. Consequently, all the components of the vector **s** would be zero. Therefore, the null hypothesis is met if the condition (or constraint) **s** = 0 is satisfied. Conversely, the alternative hypothesis *H*_*a*_ is met if the condition **s** ≠ 0 is satisfied. Thus, we can write

$$H_{0}:s=0$$ 8

$$H_{a}:s\neq 0$$ 9

Under the imposed constraints, **s** = 0, the constrained maximum likelihood estimate is obtained by

$$\begin{array}{l} {\mathrm{\theta ̂}}_{d}=\arg\;\max_{\theta_{d}}\;\Phi_{d}\left(Y|\theta_{d}\right)\\ s.t\;s=0 \end{array}$$ 10

The maximization problem of eq 10 under constraints **s** = 0 yields **θ̂**_*d*_ that is equal to the unconstrained maximum likelihood estimate (see eq 3), θ_1_, that is θ̂_1,*i*_ = θ̂_1_ ∀ *i* = 1,..., *N*. At the constrained maximum likelihood estimates, the following equations are satisfied:

$$\begin{array}{r} \nabla \Phi_{d}\left(Y|{\mathrm{\theta ̂}}_{d}\right)+\nabla s\mathrm{\alpha ̂}=0\\ s=0 \end{array}$$ 11

where **α̂** is the *N* – 1-dimensional vector of the Lagrange multipliers associated with the constraints **s**. Given that the null hypothesis holds, Aitchison and Silvey^36^ and Silvey^29^ proposed a ξ_1_ statistic that is assumed to be asymptotically distributed as a χ^2^ distribution with DoF = *N* – 1, which is equal to the number of constraints. The ξ_1_ statistic is given by

$$\xi_{1}=\mathrm{\alpha ̂}\nabla s^{T}H_{d}^{-1}\nabla s\mathrm{\alpha ̂}∼\chi_{N-1}^{2}$$ 12

where **H**_*d*_ is the *N* × *N* expected Fisher Information Matrix that is evaluated at ***θ̂***_*d*_:

$$H_{d}=\overset{N}{\underset{i=1}{\sum }}\nabla {ŷ}_{i}\left({\mathrm{\theta ̂}}_{1,i}\right)\Sigma_{y}^{-1}\nabla {ŷ}_{i}{\left({\mathrm{\theta ̂}}_{1,i}\right)}^{T}$$ 13

Note that one does not have to solve eq 10 to calculate the ξ_1_ statistic; however, ξ_1_ can be calculated as a function of the log-likelihood function evaluated at ***θ̂***_*d*_:

$$\xi_{1}=\nabla \Phi_{d}{\left(Y|{\mathrm{\theta ̂}}_{d}\right)}^{T}H_{d}^{-1}\nabla \Phi_{d}\left(Y|{\mathrm{\theta ̂}}_{d}\right)∼\chi_{N-1}^{2}$$ 14

The above formulation (eq 14) is convenient since the calculation of the Lagrange multiplier vector **α̂** is not required. Consequently, Quaglio et al.^28^ proposed that the illustrated procedure for the calculation of ξ is repeated for all the associated parameters of the model, that is ξ_*i*_ ∀ *i* = 1,..., *N*_θ_. Hence, we can obtain a heuristic measure of model misspecification, the Model Modification Index (MMI) that is given by

$${\mathrm{MMI}}_{i}=\frac{\xi_{i}}{\chi_{N-1}^{2}\left(95\%\right)}\forall i=1,...,N_{\theta }$$ 15

The MMI is the ratio between the ξ statistic and the 95% χ^2^ distribution with *N* – 1 DoF. The MMI metric aids in assessing the null hypothesis, that is, whether the parameters are state independent constants. The MMI is valuable as it can quantify the expected improvement in the log-likelihood function given a relaxation in the constraints, **s** = 0. MMI_*i*_ values above 1 signify that the null hypothesis is not true, and therefore replacing a parameter, θ_*i*_, under consideration with a state dependent function should improve the goodness-of-fit of the simulated data against the experimental data set. However, if MMI_*i*_ is below 1, then we cannot justify the replacement of θ_*i*_ with a function. We refer the reader to the original work of Quaglio et al.^28^ for a more detailed description of the Lagrange multiplier test.

### Chromatography Modeling

The Equilibrium Dispersive Model (EDM) assumes instantaneous mass transfer, extremely fast adsorption and desorption, fast convection, and slow dispersion.^11^ Since mass transfer is extremely fast, the mass transfer resistances can be lumped into an apparent dispersion coefficient. The model for a single analyte (component) consists of a mass balance that reads:

$$\frac{\partial C_{m}}{\partial t}+F\frac{\partial q}{\partial t}+u_{int}\frac{\partial C_{m}}{\partial x}=D_{app}\frac{\partial^{2}C_{m}}{\partial x^{2}},0<x<L$$ 16

where *C*_*m*_ and *q* are the concentrations of the analyte in the (bulk of the) mobile and stationary phases, respectively, *F* is the volumetric phase ratio between the stationary and the mobile phases, *u*_*int*_ is the hypothetical interstitial velocity, *D*_app_ is the apparent dispersion coefficient, and *L* is column length. The volumetric phase ratio is given by *F* = (1 – ϵ_*t*_)/ϵ_*t*_ where ϵ_*t*_ is the total porosity of the column; the hypothetical interstitial velocity is given by *u*_int_ = *Q*/*S ϵ*_*t*_, where *Q* is the volumetric flow rate, and *S* is the cross-sectional area of the column. The apparent dispersion coefficient, $D_{\mathrm{app}}$ , can be estimated employing fluid dynamics equations, but in this work we will use an approximation based on the number of theoretical plates *N*_*p*_ of the column, a parameter that can be obtained experimentally:^10,11,37^

$$D_{\mathrm{app}}=\frac{u_{\mathrm{int}}L}{2N_{p}}$$ 17

Note that eq 17 is a simplification of the original equation found in Katsoulas et al.^11^ since the original expression also depends on the isotherm. However, in this work we assume that the effect of $D_{app}$ is negligible, and thus we only seek an approximation of the apparent dispersion.

At the column inlet (*x* = 0), a Danckwerts boundary condition is adopted:

$$C_{\mathrm{in}}=C_{m}(x=0,t)-\frac{D_{\mathrm{app}}}{u_{\mathrm{int}}}\frac{\partial C_{m}(x=0,t)}{\partial x}$$ 18

where *C*_in_ is the loading concentration at the column inlet, which is initially zero, and when the sample is injected (at *t* = *t*_inj_), *C*_in_ is equal to the concentration in the sample, *C*_in_^★^. The feed pulse lasts for *V*/*Q* min and then *C*_in_ is brought back to 0. In summary:

$$C_{in}=\{\begin{array}{l} 0,\mathrm{if}\;\;t<t_{inj}\\ C_{in}^{★},\mathrm{if}\;\;t\geq t_{inj}\mathrm{and}t\leq t_{inj}+V/Q\\ 0,\mathrm{if}\;\;t>t_{inj}+V/Q \end{array}$$ 19

At the column outlet (*x* = *L*), the boundary condition reads:

$$\frac{\partial C_{m}\left(x=L,t\right)}{\partial x}=0$$ 20

The outlet boundary condition assumes that the concentration at the outlet equals the concentration immediately before the outlet.^11^

Since the mass transfer process between the mobile and stationary phases are assumed to be extremely fast, the concentration of the analyte in the bulk of the mobile phase is assumed to be in equilibrium with the concentration of the analyte in the stationary phase. Therefore, an algebraic equation is required to establish a relation between the two concentrations, known as the adsorption isotherm:

$$q=f\left(C_{m}\right)$$ 21

and is the functional relationship we seek to establish using the proposed procedure.

## Case Studies

In the following, we will illustrate the application of the diagnostic procedure, proposed in the Methodology section, based on isotherm models commonly used for the simulation of elution profiles in chromatographic columns. We will consider three distinct case studies. Cases A and B start from an approximated model (different in each case) and arrive at the “ground truth” model via modification. The ground truth represents the true underlying physics of a process. When considering in-silico experimentation, the ground truth model is the one used to generate the in-silico experiments. In Case C, however, none of the commonly used isotherm models in chromatography can accurately simulate the experimental data, and we therefore resort to using a polynomial, which via a series of modifications is able to accurately capture the underlying physics of adsorption of the system under consideration, finally matching the experimental elution profiles. In other words, in Cases A and B, we modify existing approximate models, whereas in Case C, we construct a model from scratch, assuming no prior knowledge of the isotherm.

In lieu of real experiments, in-silico experiments are used for all case studies. Using in-silico experiments rather than real experiments means we have complete knowledge of the system and can therefore accurately evaluate the appropriateness of the procedure, which would obviously not be possible with real experiments, as the adsorption isotherm would be unknown. The case studies consider the chromatogram for a single component in Reversed-Phase Liquid Chromatography (RPLC) and assume the same experimental setup, the parameters of which are given in Table 1. As the chromatograms were generated in-silico, we introduced Gaussian distributed measurement noise of 1% for each generated measurement point. The value of the measurement noise is an educated guess based on common practice in the field.^38^

**Table 1:** Experimental Setup Used for All Case Studies

**parameter**	**symbol**	**value**	**unit**
column length	*L*	15	cm
inner diameter	*D*	0.46	cm
total porosity	ϵ_*t*_	0.635	-
apparent dispersion coefficient	$D_{\mathrm{app}}$	0.006	cm^2^/min

For the simulation and maximum likelihood parameter estimation, we used gPROMS ModelBuilder.^39^ The grid was discretized into 100 elements using the first-order backward finite differences method. Parameter estimation was solved via the NLPMSO solver, which is a multistart algorithm that samples the parameter space through a Sobol sequence^40^ to construct sets of initial guesses for the parameters, **θ**. The Sobol sequence considers lower and upper bounds (set by the modeler) of the parameters as uniform distributions and produces initial guesses that are distributed as evenly as possible in the parameter space. NLPMSO next solves an optimization problem employing the default solver, NLPSQP, for each of the sets of initial guesses and updates the best solution. For the following case studies, we set the number of initial guess points to 10 in order to balance computational efficiency and optimality. As highlighted later in the paper, we increased the numbers of initial points when appropriate. For an optimization run comprising 10 local searches, the CPU time varied between approximately 320 s (∼5.3 min) and 940 s (∼15.6 min). When the number of local searches was increased to, for instance, 50, the total computation time rose to as much as 3400 s on an Intel Core i7–1185G7 processor with 16 GB of RAM, while employing the gPROMS parallel computation license.

### Case A – Modulated Langmuir Isotherm

One of the most commonly employed techniques to optimize RPLC chromatographic separations^4^ is to take into account a varying mobile phase composition. Therefore, the commonly employed Langmuir isotherm can be modulated according to the Linear Solvent Theory (LSS) to capture the effect of the fraction of the organic modifier of the solvent. There are two prevailing models, the first assuming that the saturation capacity, *q*_sat_, is not affected by changes in the solvent composition,^10,15^ where the LSS modulated Langmuir model reads:

$$q(C_{m},\phi )=\frac{K\exp(-S_{a}\phi )C_{m}}{1+\frac{K}{q_{\mathrm{sat}}}\exp(-S_{a}\phi )C_{m}}$$ 22

where *K* is the retention factor at infinite dilution (i.e., at φ = 0), *q*_*sat*_ is the saturation capacity, *S*_*a*_ is the solvent strength parameter, and φ is the fraction of the organic modifier of the solvent. (Note that for the sake of simplicity we will be referring to *K* as the retention factor.) The second option assumes that the saturation capacity depends on the fraction of the organic modifier,^19,41^ and reads:

$$q(C_{m},\phi )=\frac{K\exp(-S_{a}\phi )C_{m}}{1+\frac{K}{q_{\mathrm{sat}}}\exp(-S_{b}\phi )C_{m}}$$ 23

where *S*_*b*_ is the solvent strength parameter associated with the effect of the solvent on the saturation capacity. Case A considers that both the retention factor and the saturation capacity parameters depend on the solvent composition, and thus the “real” system described by in-silico model is the EDM coupled with eq 23, with *K* = 118.58 (−), *q*_*sat*_ = 34.45 (mg/mL), *S*_*a*_ = 9.36 (−), and *S*_*b*_ = 7.5 (−). These parameter values were determined by considering the typical orders of magnitude reported in the literature for the same parameters and by performing simulations to assess whether the resulting chromatograms were reasonable. In the following, we will consider eq 22 as the proposed, or approximated, model and eq 23 as the actual (or ground truth) system model.

The experimental design vector consists of two degrees of freedom, the sample volume, *V*, and the fraction of the organic modifier, ϕ, that is, **φ** = [*V*, ϕ], i.e. these are the variables that can be manipulated in the system. For a time interval of *V*/*Q* min (starts at *t* = 0), the inlet concentration jumps from *C*_in_ = 0 to *C*_in_ ≡ *C*_in_^★^ = 5 mg/mL and then returns back to 0. Initially, we employ five experiments, at conditions prescribed solely by process intuition and not by any particular design of experiments (DoE), in order to generate few but informative experiments. (One might argue that a better solution would be to design those experiments with a conventional DoE method such as latin hypercube sampling (LHS), or Sobol sampling (SS). Our aim was to design as few experiments as possible, but at the same time informative enough to aid in an effortless parameter estimation. Therefore, DoE methods that produce pseudorandom samples such as LHS and SS do not always produce informative experiments at small number of samples.) The experimental conditions are given in Table 2.

**Table 2:** Case A: Experimental Conditions

		**experiment no.**				
**control variable** (*C*_in_^★^ = 5 mg/mL, *Q* = 1 mL/min)	**symbol**	**1**	**2**	**3**	**4**	**5**
sample volume	*V* (mL)	0.5	0.8	1	1	1.2
fraction of organic modifier	ϕ (−)	0.1	0.2	0.3	0.4	0.5

After performing the experiments at the prescribed conditions of Table 2, we obtained the corresponding chromatograms. To start the modeling work, the modeler must first propose a likely or approximate adsorption isotherm model for the system under consideration (since for a real system the modeler would not know the underlying ground truth model), and then use the initial experiments to estimate the associated parameter values. In the following, we assume that the modeler has proposed the modulated Langmuir isotherm model of eq 22 as the approximated model in order to perform parameter estimation. By maximizing the log-likelihood function (eq 3), we obtained the vector of maximum likelihood parameter estimates, ***θ̂***, reported in Table 3, second row.

**Table 3:** Case A: Maximum Likelihood Parameter Estimates of the Approximated and Modified Models

	**maximum likelihood estimates**			
**model structure**	***K***	***q*_sat_**	***S_a_***	***S_b_***
**ground truth values**	118.58	34.45	9.36	7.50
approximated model (eq 22)	122.14	27.94	9.58	–
modified model (eqs 23, 24)	118.60	34.44	9.36	7.50

The corresponding goodness-of-fit test, conducted by the χ^2^ test, is reported in Table 4 (top row). The χ^2^ test for the assumed model (top row), demonstrates a significant mismatch between the simulated and experimental data, and the goodness-of-fit test has clearly failed. Therefore, we now follow the diagnostic procedure to examine whether any of the parameters allow for modification.

**Table 4:** Case A: Goodness-of-Fit Test of the Approximated and Modified Models

	**goodness-of-fit test**		
**model structure**	**χ^2^ (95%)**	**χ_*c*_^2^**	**outcome**
approximated model (eq 22)	388,340	681	failed
modified model (eqs 23, 24)	639	681	passed

The three MMI values associated with the parameters of the approximated model are reported in Table 5 (top row). All three values are greater than 1, thus all failing the Lagrange multiplier test. However, looking closely, the MMI value of the *q*_sat_ parameter is an order of magnitude larger than the MMI values for the other two parameters; therefore, *q*_sat_ constitutes a good modification candidate. Hence, we propose to replace *q*_sat_ with *q*_sat_ = *q*_sat_ exp­(−*S*_*c*_ϕ), since there could be a potential relation between the solvent composition and the saturation capacity, thereby turning it from a constant parameter to a function of the state variables. Note that the selection procedure for the modification at this stage is neither random nor based on trial and error; rather, it relies on the modeler’s good knowledge and physical understanding of the process. Therefore, we modify the approximated model as follows:

$$\begin{array}{ll} q(C_{m},\phi ) & =\frac{K\exp(-S_{a}\phi )C_{m}}{1+\frac{K}{q_{\mathrm{sat}}}\exp(-S_{a}\phi )C_{m}}\overset{q_{\mathrm{sat}}=q_{\mathrm{sat}}\exp(-S_{c}\phi )}{\to }\\ q(C_{m},\phi ) & =\frac{K\exp(-S_{a}\phi )C_{m}}{1+\frac{K}{q_{\mathrm{sat}}\exp(-S_{c}\phi )}\exp(-S_{a}\phi )C_{m}}\to \\ q(C_{m},\phi ) & =\frac{K\exp(-S_{a}\phi )C_{m}}{1+\frac{K}{q_{\mathrm{sat}}}\exp[(-S_{a}+S_{c})\phi ]C_{m}}\overset{S_{b}=-(S_{c}-S_{a})}{\to }\\ q(C_{m},\phi ) & =\frac{K\exp(-S_{a}\phi )C_{m}}{1+\frac{K}{q_{\mathrm{sat}}}\exp(-S_{b})\phi C_{m}} \end{array}$$ 24

**Table 5:** Case A: Model Modification Index (MMI) for the Associated Parameters of the Approximated and Modified Models

	**MMI**			
**model structure**	***K***	***q*_sat_**	***S*_*a*_**	***S*_*b*_**
approximated model (eq 22)	2837	19,709	2837	-
modified model (eqs 23, 24)	0.40	0.31	0.36	0.31

The outcome of the modification (as expected based on the in-silico model) results in eq 24, which is identical to the in-silico model of eq 23. Repeating the parameter estimation with the now modified model leads to simulated profiles that accurately match the experimental profiles, corroborated by the χ^2^ values that are now within the acceptable range (Table 4, bottom row). The mismatch between the approximated model and the experiments has now been compensated by the modified model, as observed for instance at the chromatogram of experiment no. 4 in Figure 2. The maximum likelihood estimates of the two models, approximated and modified, are reported in Table 3.

**Figure 2:**
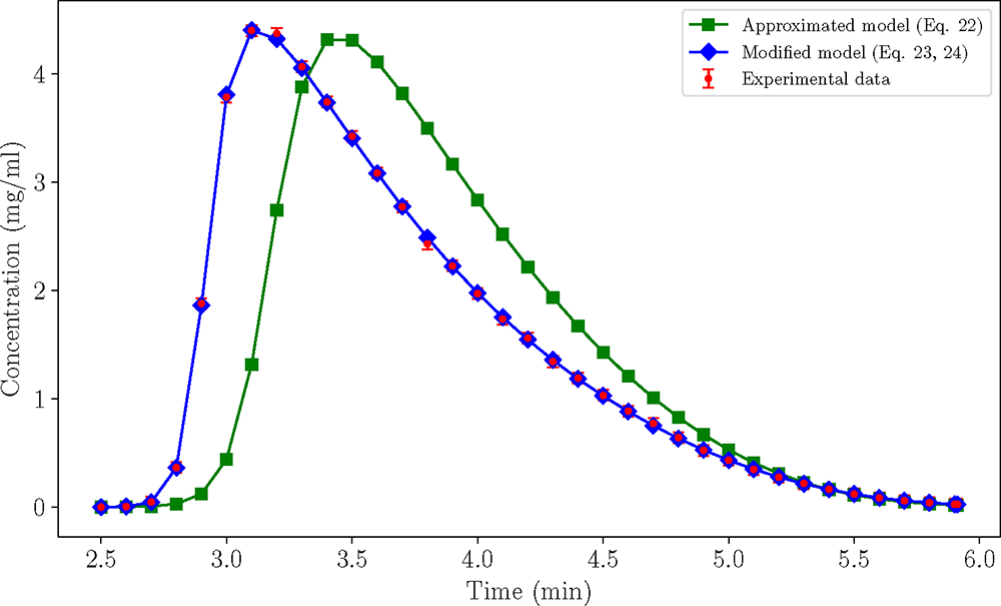
Case A: Comparison between the chromatograms produced by the approximated and the modified models at conditions prescribed by experiment no. 4 (*V* = 1 mL, ϕ = 0.4).

Finally, we calculate the MMI values for the associated parameters of the modified model (Table 5, bottom row). The values now rest well below 1, thus rendering the new model structure acceptable for further model activity. The radar charts of Figure 3 visualize the value progression of the MMI from the approximated model to the modified model, where the values turned from completely asymmetric to symmetric, respectively.

**Figure 3:**
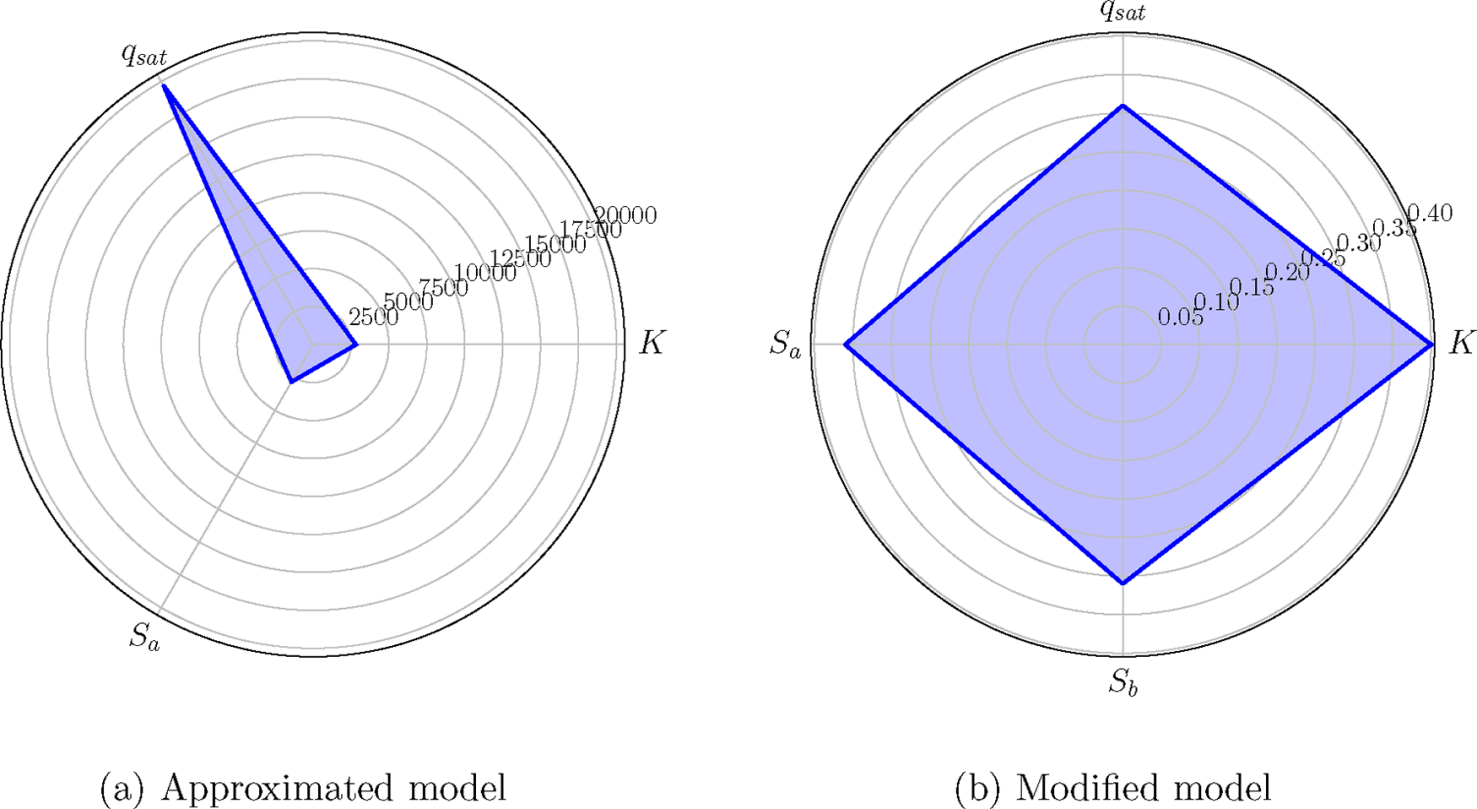
Case A: Radar chart of the MMI values associated with the parameters of the (a) approximated model and (b) the modified model.

### Case B – Quadratic Relationships in Modulated Langmuir Isotherm

Modelers in the field of chromatography consider the LSS theory widely acceptable as a means to capture the effects of the solvent composition on the isotherm parameters.^42^ It is straightforward for low concentration systems to obtain a trend between, for instance, the Henry coefficient of the linear isotherm and the fraction of the organic modifier, by measuring the retention time of Gaussian chromatographic peaks.^4,37^ But when one needs to estimate parameters for systems that only appear in overloaded conditions, finding a trend between those parameters and the solvent composition might be more intricate. First, one would have to propose a trend, e.g. LSS, and then via curve-fitting ascertain if the model can capture the assumed trend. Candidate models for separation at different solvent composition conditions usually involve the above-mentioned modulated Langmuir isotherms, eqs 22 and 23, which assume the LSS theory. There are, however, cases where a quadratic function might better explain the parameter dependence on solvent composition.^42–44^ If only the saturation capacity, *q*_sat_, depends quadratically on the organic modifier, the modulated Langmuir reads as

$$q(C_{m},\phi )=\frac{K\exp(-S_{a}\phi )C_{m}}{1+\frac{K}{q_{\mathrm{sat}}}\exp(-S_{b}\phi +S_{c}\phi^{2})C_{m}}$$ 25

where *S*_*c*_ is also a solvent strength parameter. We employed the model of eq 25 as the ground truth model, and generated in-silico experiments based on conditions given in Table 6, which slightly differ in relation to the conditions used in Case A in order to demonstrate the robustness of the methodology across varying conditions.

**Table 6:** Case B: Experimental Conditions

		**experiment no.**				
**control variable (*C*_in_^★^ =** 5 mg/mL, *Q* = 1 mL/min)	**symbol**	**1**	**2**	**3**	**4**	**5**
sample volume	*V* (mL)	0.5	0.8	1	1.2	1.2
fraction of organic modifier	ϕ (−)	0.1	0.2	0.3	0.5	0.3

After generating the in-silico experiments, we performed a parameter estimation with the proposed approximated model of eq 23 only to find that there is a mismatch between the simulated (at maximum likelihood estimates, **θ̂**, reported in Table 7) and the experimental chromatograms, which was corroborated by the very large χ^2^ values in Table 8, eventually resulting in the approximated model failing the χ^2^ test.

**Table 7:** Case B: Maximum Likelihood Parameter Estimates of the Approximated and Modified Models

	**maximum likelihood estimates**				
**model structure**	***K***	***q*_sat_**	***S*_*a*_**	***S*_*b*_**	***S*_*c*_**
**ground truth values**	118.58	34.45	9.36	3.10	4.20
approximated model (eq 23)	118.90	37.87	9.41	1.84	–
modified model (eqs 25, 26, 27)	118.55	34.45	9.36	3.10	4.21

**Table 8:** Case B: Goodness-of-Fit Test of the Approximated and Modified Models

	**goodness-of-fit test**		
**model structure**	**χ^2^ (95%)**	**χ_*c*_^2^**	**outcome**
approximated model (eq 23)	76,374	791	failed
modified model (eqs 25, 26, 27)	691	791	passed

Next, the MMI for each of the associated parameters of the approximated model was calculated, given in Table 9, to conduct the Lagrange multiplier test. All of the parametric MMI values are much greater than 1, and thus the model fails the Lagrange multiplier test. Two of the MMI values stand out, observed also on the radar charts of Figure 5, those of *q*_sat_ and *S*_*b*_, suggesting that these parameters could be potential functions of the state variables. Although the MMI of the two parameters are of similar order of magnitude, we first proceeded with modifying the parameter with the largest associated MMI value, that is *S*_*b*_. *S*_*b*_ can potentially be replaced with a function of ϕ, i.e. *S*_*b*_ = *S*_*b*_ + *S*_*c*_ϕ. This is a simple function that assumes that *S*_*b*_ is not constant but rather a linear function of the organic modifier. Substituting the said function in the approximated model of eq 23 gives

$$\begin{array}{ll} q(C_{m},\phi ) & =\frac{K\exp(-S_{a}\phi )C_{m}}{1+\frac{K}{q_{\mathrm{sat}}}\exp(-S_{b}\phi )C_{m}}\overset{S_{b}=S_{b}+S_{c}\phi }{\to }\\ q(C_{m},\phi ) & =\frac{K\exp(-S_{a}\phi )C_{m}}{1+\frac{K}{q_{\mathrm{sat}}}\exp[(-S_{b}-S_{c}\phi )\phi ]C_{m}}\to \\ q(C_{m},\phi ) & =\frac{K\exp(-S_{a}\phi )C_{m}}{1+\frac{K}{q_{\mathrm{sat}}}\exp(-S_{b}\phi -S_{c}\phi^{2})C_{m}} \end{array}$$ 26

It is also interesting to instead examine potential modifications in *q*_sat_, and evaluate the corresponding modified model. Thus, we again start from eq 23 and replace *q*_sat_ with *q*_sat_ = *q*_sat_ exp­(−*S*_*c*_ϕ^2^):

$$\begin{array}{ll} q(C_{m},\phi ) & =\frac{K\exp(-S_{a}\phi )C_{m}}{1+\frac{K}{q_{\mathrm{sat}}}\exp(-S_{b}\phi )C_{m}}\overset{q_{\mathrm{sat}}=q_{\mathrm{sat}}\exp(-S_{c}\phi^{2})}{\to }\\ q(C_{m},\phi ) & =\frac{K\exp(-S_{a}\phi )C_{m}}{1+\frac{K}{q_{\mathrm{sat}}\exp(-S_{c}\phi^{2})}\exp(-S_{b}\phi )C_{m}}\to \\ q(C_{m},\phi ) & =\frac{K\exp(-S_{a}\phi )C_{m}}{1+\frac{K}{q_{\mathrm{sat}}}\exp(-S_{b}\phi +S_{c}\phi^{2})C_{m}} \end{array}$$ 27

Note that the two distinct modifications resulted in models that are equivalent, since only the sign of *S*_*c*_ is different between eqs 26 and 27. Moreover, eq 27 is, not surprisingly, identical to the ground truth model of eq 25. The outcome of the two modifications was somewhat anticipated, since the MMI values alluded to a modification in the denominator of the original approximated model.

**Table 9:** Case B: Model Modification Index (MMI) for the Associated Parameters of the Approximated and Modified Model

	**MMI**				
**model structure**	*K*	*q*_sat_	*S*_*a*_	*S*_*b*_	*S*_*c*_
approximated model (eq 23)	350	4231	1198	4329	-
modified model (eqs 25, 26, 27)	0.45	0.7	0.66	0.86	0.37

eq 27 is carried forward for a new round of parameter estimation against the same initial in-silico experiments of Table 6. The modified model simulates the experimental data accurately and passes the χ^2^ test as reported in Table 8 (bottom row). The modified model reduced the mismatch between the initial approximated model and the experiments, as observed for instance at the chromatogram of experiment no. 4 in Figure 4. The optimal estimates of both the approximated and modified models are reported in Table 7 (bottom row). Although the final MMI values are not entirely symmetric according to the radar chart of Figure 5, all of them rest below 1 (Table 9, bottom row), thus successfully passing the Lagrange multiplier test.

**Figure 4:**
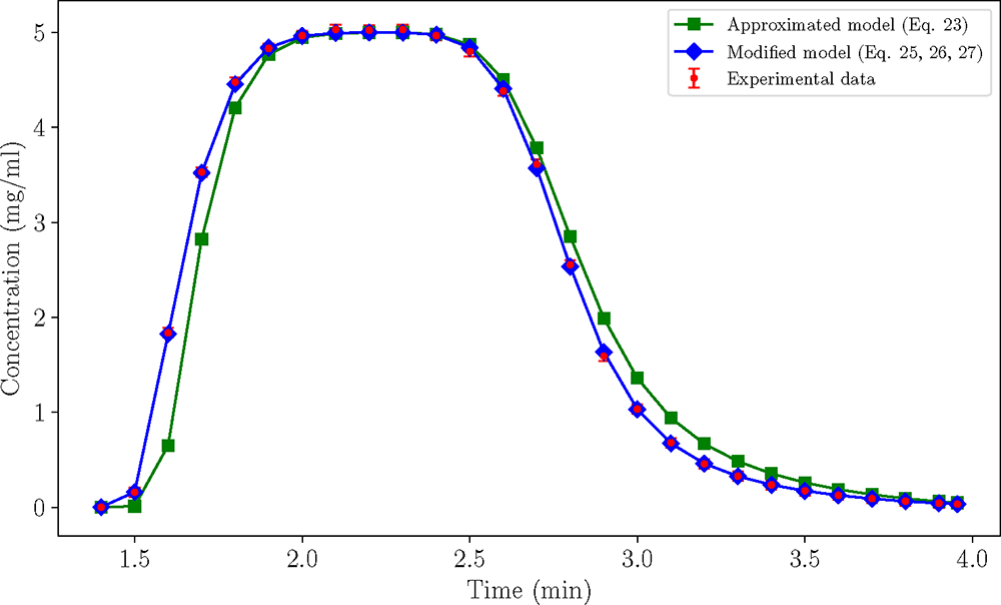
Case B: Comparison between the chromatograms produced by the approximated and the modified models at conditions prescribed by experiment no. 4 (*V* = 1.2 mL, ϕ = 0.5).

**Figure 5:**
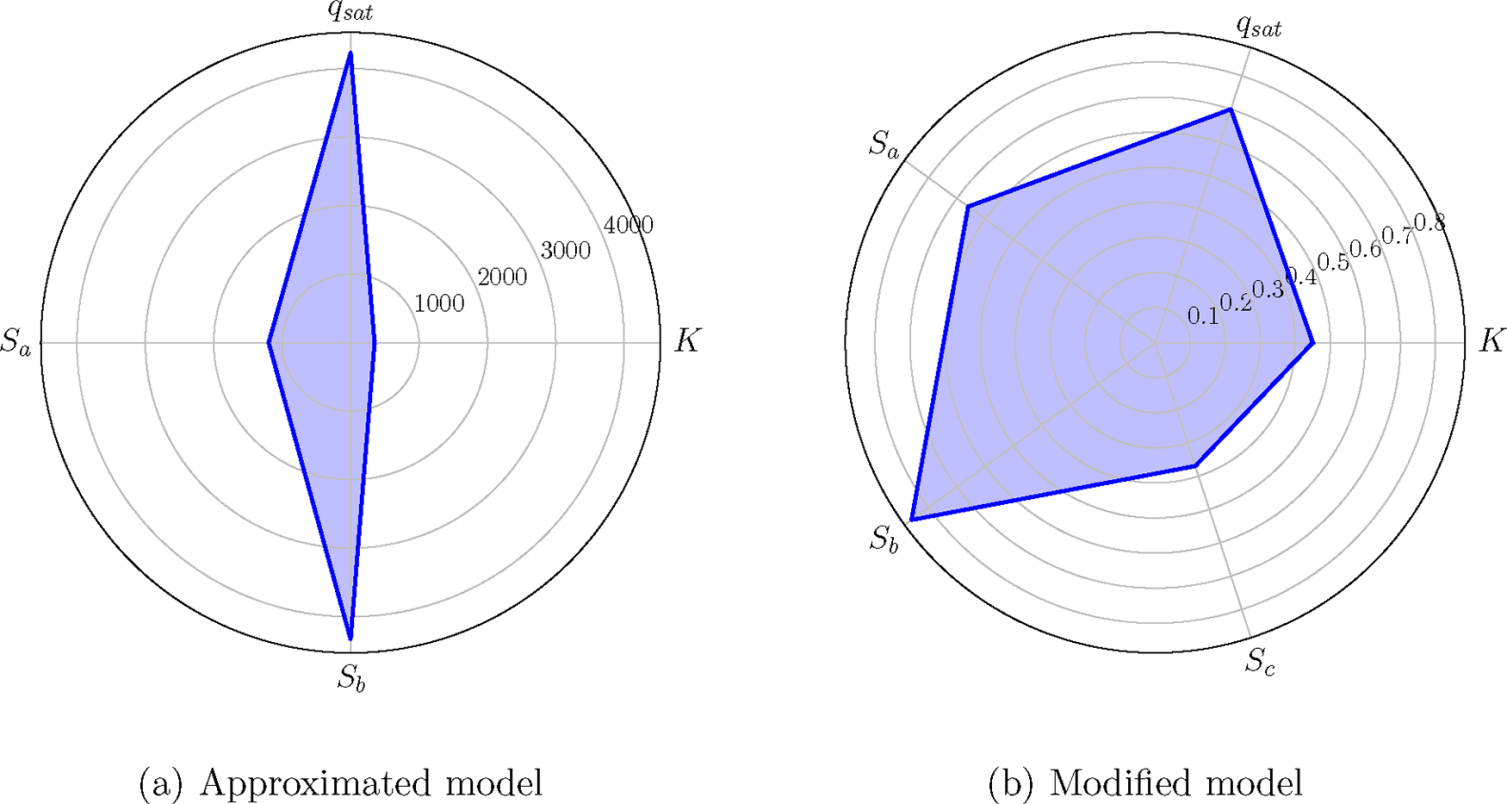
Case B: Radar chart of the MMI values associated with the parameters of the (a) approximated model and (b) the modified model.

### Case C – Replacement with a Beyond-Isotherm Model

Cases A and B explored how the proposed diagnostic procedure could aid in obtaining an accurate model by identifying individual parameters that were not constants and replacing them with functions. The procedure relied on the modeler proposing models that, while potentially imperfect, offered a reasonable approximation to reality, allowing the diagnostic procedure to refine and identify better models. When proposing models, a modeler aims to explain the underlying physical and chemical phenomena with good enough mathematical approximations; and there will inevitably be models that are more accurate than others. For chromatography, isotherm models often share a similar model structure because they are based on physics described by first principles. Case C will explore potential remedies in the case that none of the known isotherms are able to simulate the experiments accurately.

Case C considers a quadratic isotherm^14,45,46^ to produce the in-silico experiments:

$$q\left(C_{m}\right)=\frac{q_{sat}\left(bC_{m}+2b^{\prime }C_{m}^{2}\right)}{1+bC_{m}+b^{\prime }C_{m}^{2}}$$ 28

where *b* and *b′* are retention factor parameters. The aim of this particular case study is to consider an isotherm that is rarely proposed in model identification procedures and does not clearly produce a common, single type of chromatogram. The quadratic isotherm is an excellent choice due to its complex nature, which at low concentrations produces Langmuir-type (tailing) peaks because of the dominating first-order term, while at higher concentrations its peaks demonstrate anti-Langmuirian behavior (fronting), depicted in Figure 6.

**Figure 6:**
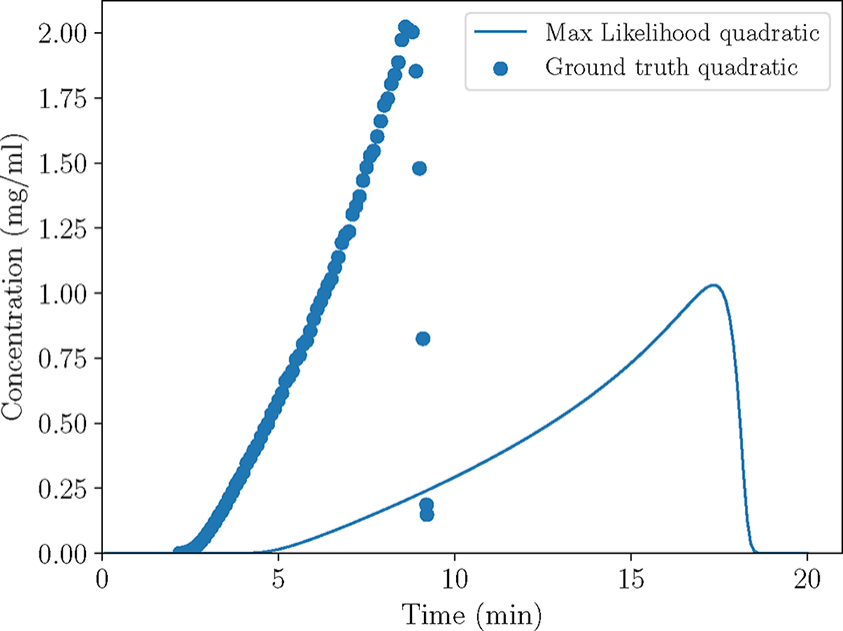
Case C: Comparison between the chromatograms produced by the quadratic isotherm based on the maximum likelihood estimate and the ground truth quadratic isotherm (*V* = 2 mL, *C*_in_^★^ = 6 mg/mL, *Q* = 2 mL/min).

Case C involved only experiments under isocratic conditions and capitalized on leveraging the sample volume, *V*, the sample inlet concentration *C*_in_^★^, and the flow rate, *Q*. We designed eight experiments via a full factorial design at the corners of the design space, that is *V* = {0.5, 2} ml, *C*_in_^★^ = {1, 6} mg/mL, *Q* = {0.5, 2} mL/min (Figure 7) and the experiments were executed at the given conditions. Next, we proposed three candidate isotherm models to proceed with the parameter estimation:

1. the Langmuir isotherm:^4^
$$q\left(C_{m}\right)=q_{sat}\frac{bC_{m}}{1+bC_{m}}$$ 29
  where the retention factor is $b=\frac{K}{q_{\mathrm{sat}}}$ ,
2. the second-order Langmuir–Freundlich isotherm:^5^
$$q\left(C_{m}\right)=q_{sat}\frac{{\left(bC_{m}\right)}^{2}}{1+{\left(bC_{m}\right)}^{2}}$$ 30
3. and, last, the quadratic ground truth model, that is the quadratic isotherm of eq 28 using parametric values of Table 10.

**Figure 7:**
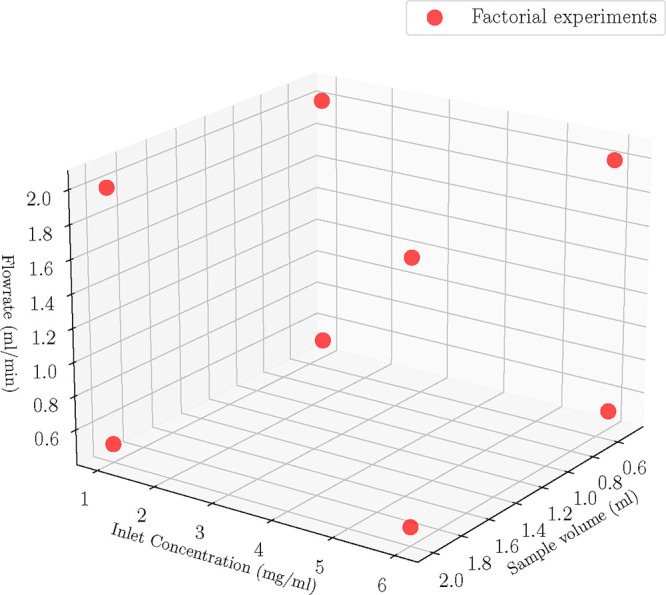
Case C: Full factorial generated experimental conditions used for parameter estimation.

**Table 10:** Case C: Ground Truth Values of the Quadratic Isotherm Model

	**ground truth values**		
**model structure**	***b* (mL/mg)**	***b′* (mL/mg)**	***q*_sat_** (mg/mL)
quadratic (eq 28)	0.04	0.02	123.2

Note that eqs 28 and 30 are commonly expressed with *b* parametrization in the literature, and therefore we kept the same parametrization for the Langmuir isotherm of eq 29 for this case study (*b* instead of *K*).

The Langmuir and Langmuir–Freundlich isotherms with the maximum likelihood parameter estimates failed to recreate the experiments (see Table 11 for χ^2^ values), not only because they failed the χ^2^ test by a large margin, but also because the trends they produce are not representative of the process, an outcome highly anticipated since there are fundamental differences between the candidate models and the ground truth model.

**Table 11:** Case C: Goodness-of-Fit Test of the Approximate Models

	**goodness-of-fit test**		
**model structure**	**χ^2^ (95%)**	**χ_*c*_^2^**	**outcome**
quadratic (eq 28)	8.38 × 10^6^	968.83	failed
Langmuir (eq 29)	7.37 × 10^6^	969.87	failed
Langmuir–Freundlich (eq 30)	8.57 × 10^6^	969.87	failed

The chromatogram produced by the quadratic isotherm with parameters from the initial maximum likelihood estimates show an offset of the peak width relative to the experiments, thus is failing to recreate the experiments even though the isotherm model is identical to that of the ground truth model (Figure 6). This is quite an unexpected outcome since the two model structures are identical, yet the maximum likelihood estimates are poor. To potentially alleviate the mismatch, we re-executed the experiments three times, also increasing the NLPMSO initial points. Since the mismatch did not improve, this pointed to a potentially problematic objective function. The most likely explanation for this result is that the log-likelihood objective function is flat with respect to the model parameters. Hence, we need to employ a different strategy toward finding a model structure that is not only representative of the process, but also has estimable parameters.

Most known isotherms, if expanded around zero using a Taylor series, result in a polynomial function. A polynomial therefore makes a good candidate to replace classic isotherm structures. Note, however, that a fundamental downside of using a polynomial is that it lacks interpretability in relation to an isotherm. Isotherm parameters are physical quantities whose initial guesses can be potentially retrieved from the literature or from previous experience. For instance, the *q*_sat_ parameter denotes where the concentration in the stationary phase stops increasing with an increase in the mobile phase concentration. Conversely, polynomial parameters have no physical meaning. However, simple polynomials, such as the one used in this case, have an interpretable model structure that can inform modelers about, for instance, the curvature of the isotherm. Hence, the polynomial approach still preserves some interpretability vis-a-vis black-box models that tend to be very complex and lack interpretability.

We first proposed a second-degree polynomial:

$$q\left(C_{m}\right)=a_{1}C_{m}+a_{2}C_{m}^{2}$$ 31

where *a*_*i*_ are the polynomial parameters. Note that since for *C*_*m*_ = 0, it must be *q* = 0 (that is, the stationary phase concentration that equilibrate a zero mobile phase concentration must be zero), in the polynomial the parameter *a*_0_ must be zero. Next, we executed a new parameter estimation using the new candidate model against the full factorial experiments (see Figure 7), and then evaluated the χ^2^ test (see Table 12, first row). Since the χ^2^ test failed, we had to evaluate the MMI of the associated polynomial parameters to assess which of the parameters should be further modified to improve the goodness-of-fit. According to Table 13, the MMI value for *a*_2_ is much greater than 1, and 1 order of magnitude larger than the corresponding MMI value for *a*_1_. Therefore, we proposed replacing *a*_2_ with *a*_2_ = *a*_2_ + *a*_3_ *C*_*m*_, thereby arriving at a third-degree polynomial:

$$q\left(C_{m}\right)=a_{1}C_{m}+a_{2}C_{m}^{2}+a_{3}C_{m}^{3}$$ 32

By repeating the parameter estimation and assessing the χ^2^ test, we found that, although the χ^2^ value dropped significantly from the considerable value of 466,886 to 1,440 (see Table 12), the model still failed the goodness-of-fit test since the critical value χ_*c*_^2^ equals 969. In accordance with the proposed procedure, we re-evaluated the MMI values of the modified model of eq 32. The MMI values of the associated polynomial parameters decreased, yet they still violated the upper limit of 1. Therefore, we selected parameter *a*_3_, as it had an MMI value slightly higher than *a*_2_, and proposed two distinct modifications. First, we proposed replacing *a*_3_ with *a*_3_ = *a*_3_ + *a*_4_ tanh­(*C*_*m*_)*C*_*m*_^3^, which resulted in the following modified model:

$$q\left(C_{m}\right)=a_{1}C_{m}+a_{2}C_{m}^{2}+a_{3}C_{m}^{3}+a_{4}\tanh\left(C_{m}\right)C_{m}^{3}$$ 33

and second, we proposed replacing *a*_3_ with *a*_3_ + *a*_4_ *C*_*m*_, which resulted in the following modified quadratic model:

$$q\left(C_{m}\right)=a_{1}C_{m}+a_{2}C_{m}^{2}+a_{3}C_{m}^{3}+a_{4}C_{m}^{4}$$ 34

**Table 12:** Case C: Goodness-of-Fit Test of the Proposed Models

	**goodness-of-fit test**		
**model structure**	**χ^2^ (95%)**	**χ_*c*_^2^**	**outcome**
*a*_1_ *C*_*m*_ + *a*_2_ *C*_*m*_^2^ (eq 31)	466,886	970	failed
*a*_1_ *C*_*m*_ + *a*_2_ *C*_*m*_^2^ + *a*_3_ *C*_*m*_^3^ (eq 32)	1440	969	failed
*a*_1_ *C*_*m*_ + *a*_2_ *C*_*m*_^2^ + *a*_3_ *C*_*m*_^3^ + *a*_4_ tanh(*C*_*m*_)*C*_*m*_^3^ (eq 33)	1195	968	failed
*a*_1_ *C*_*m*_ + *a*_2_ *C*_*m*_^2^ + *a*_3_ *C*_*m*_^3^ + *a*_4_ *C*_*m*_^4^ (eq 34)	958	968	passed

**Table 13:** Model Modification Index (MMI) for the Associated Parameters of the Proposed Models in Case C

	**MMI**			
**model structure**	*a*_1_	*a*_2_	*a*_3_	*a*_4_
*a*_1_ *C*_*m*_ + *a*_2_ *C*_*m*_^2^ (eq 31)	1863	43,540	-	-
*a*_1_ *C*_*m*_ + *a*_2_ *C*_*m*_^2^ + *a*_3_ *C*_*m*_^3^ (eq 32)	12.24	20.94	21.57	-
*a*_1_ *C*_*m*_ + *a*_2_ *C*_*m*_^2^ + *a*_3_ *C*_*m*_^3^ + *a*_4_ tanh(*C*_*m*_)*C*_*m*_^3^ (eq 33)	6.43	10.41	10.82	10.92
*a*_1_ *C*_*m*_ + *a*_2_ *C*_*m*_^2^ + *a*_3_ *C*_*m*_^3^ + *a*_4_ *C*_*m*_^4^ (eq 34)	3.06	3.06	3.06	3.01

In lieu of increasing the polynomial degree by adding a *C*_*m*_^4^ term, we opted for the tanh­(*C*_*m*_)*C*_*m*_^4^ term to mitigate potential excessive polynomial growth. The hyperbolic tangent term can damp high-order effects, potentially offering good numerical stability and generalization. Similar damping can be achieved using other nonlinear basis functions such as sigmoid, arctangent, etc. Although eq 33 improved the predictions in relation to eq 32, the χ^2^ test was not satisfied (see Table 12). The Lagrange multiplier was also not satisfied, albeit the MMI values of the associated parameters came closer to 1. On the other hand, the fourth-degree polynomial of eq 34 improved the predictions and satisfied the χ^2^ test. In terms of the Lagrange multiplier test, the MMI values of the polynomial parameters of eq 34 dropped significantly to ≈3, yet, since the values are still above 1, they do not satisfy the test threshold. The radar chart of Figure 8 summarizes the MMI values of the modified models parameters, where we can observe the gradual decrease in the MMI values considering the third-degree polynomial and its two offsprings. Table 14 summarizes the optimal estimates of the models considered for parameter estimation in Case C.

**Figure 8:**
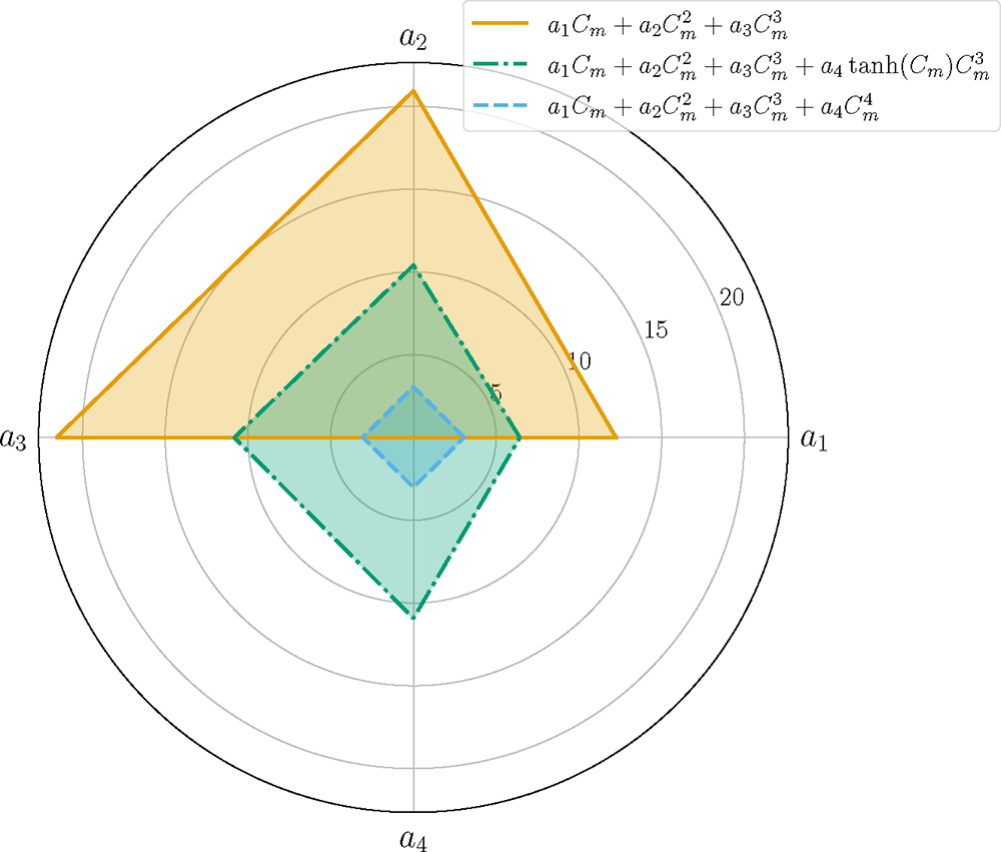
Case C: Radar chart of the MMI values of the associated parameters of the proposed polynomials.

**Table 14:** Case C: Maximum Likelihood Parameter Estimates of the Models Considered

	**maximum likelihood estimates**			
**model structure**	***a*_1_**	***a*_2_**	***a*_3_**	***a*_4_**
*a*_1_ *C*_*m*_ + *a*_2_ *C*_*m*_^2^ (eq 31)	5.05	3.75	-	-
*a*_1_ *C*_*m*_ + *a*_2_ *C*_*m*_^2^ + *a*_3_ *C*_*m*_^3^ (eq 32)	4.92	4.83	–0.46	-
*a*_1_ *C*_*m*_ + *a*_2_ *C*_*m*_^2^ + *a*_3_ *C*_*m*_^3^ + *a*_4_ tanh(*C*_*m*_)*C*_*m*_^3^ (eq 33)	4.93	4.70	–0.078	–0.35
*a*_1_ *C*_*m*_ + *a*_2_ *C*_*m*_^2^ + *a*_3_ *C*_*m*_^3^ + *a*_4_ *C*_*m*_^4^ (eq 34)	4.93	4.77	–0.38	–0.02

At this stage, modelers might encounter the following dilemma: should they continue diagnosing and modifying the model, or should they stop since the χ^2^ test is satisfied even if the MMI is not? (Note that according the proposed procedure, when the considered model passed the goodness-of-fit test, the procedure can terminate.) The most sensible choice in Case C is to accept the model and stop modifying it further, as not only do the simulations fit the experiments well based on the χ^2^ test, but also the MMI values do not point to a specific parameter that could be considered for replacement with a function since the MMI values are practically the same. Nevertheless, to show the potential impact of further modification, we also explored the outcome of a further potential replacement of one of the parameters.

The most obvious modification is to replace *a*_4_ with *a*_4_ + *a*_5_ *C*_*m*_, thereby considering a fifth-degree polynomial. Before discussing the outcome of adding more terms to the proposed polynomial, it is worth discussing the equivalence between a Taylor expansion of the ground truth model and the proposed polynomial functions. A Taylor expansion of the ground truth, or in-silico, model (eq 28) around *C*_*m*_ = 0 results in the polynomial (see the Supporting Information for the full derivation):

$$q(C_{m})=q_{\mathrm{sat}}[bC_{m}+(-b^{2}+2b^{\prime })C_{m}^{2}+(-3bb^{\prime }+b^{3})C_{m}^{3}+(4b^{2}b^{\prime }-2b^{\prime 2})C_{m}^{4}+O(C_{m}^{5})]$$ 35

The full Taylor expansion (involving an infinite number of terms) converges under *C*_*m*_ ≈ 7 mg/mL, which is the radius of convergence (see the Supporting Information for the derivation of the convergence radius value). The notion of the convergence radius is illustrated in Figure 9, where a 15th order Taylor expansion of the initial model starts diverging close to *C*_*m*_ = 7 mg/mL.

**Figure 9:**
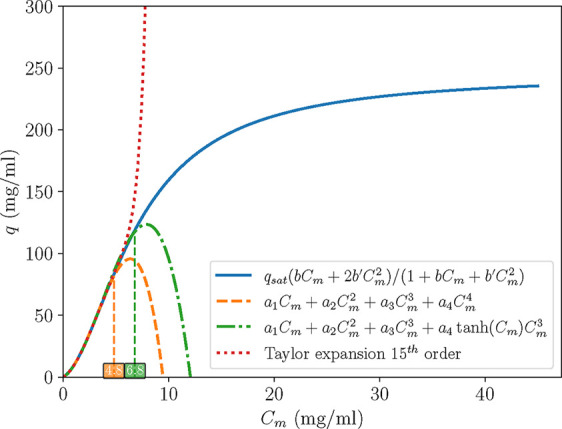
Case C: The initial isotherm (blue) vs its Taylor approximation (red) vs two approximated polynomials (orange and green).

Since in our in-silico experiments the analyte concentration is bounded by the inlet value of 6 mg/mL, adding higher order terms to the expansion would result in more accurate results. Note that the Taylor expansion results in a polynomial of specific coefficients that depend on the parameters of the true function according to eq 35, while the polynomial parameters (i.e., the coefficients) of, e.g. eq 31 through eq 34, always take the maximum likelihood parameter estimates. Now, in the case where *C*_*m*_ ≤ 7 mg/mL, the proposed polynomials are expected to be equivalent to the corresponding (truncated) Taylor expansion; hence, higher-order terms in the polynomial would render the results more accurate, but note that they cannot predict the saturation effect that most isotherms entail. Figure 9 illustrates the inability of the polynomials (eqs 32 and 33) to capture the saturation effect.

However, that (i.e., more accurate results) was not the outcome we encountered for the higher order polynomial model according to Figure 9. On the contrary, not only did the fifth-degree polynomial model fail to yield more accurate chromatograms, but it essentially produced chromatograms not representative of the process, where the peak maxima of the experiments and simulations differed by few minutes on the chromatogram. This could potentially be attributed to a flat or a highly nonconvex objective function that is prone to local optima, although we drastically increased the number of initial points of the NLPMSO solver from 10 to 40.

Although the χ^2^ test has indicated that eq 34 is a better model than eqs 31–33, we employ all of them to compare their predictions and verify the superiority of the chromatograms obtained with eq 34 within the design space, and compare it with the parent models (eqs 31 and 32), as well as the competitor model of eq 33. We therefore generated 24 new experiments sampling from a Sobol sequence,^40^ thereby ensuring quasi-randomness in the experimental conditions. The 24 Sobol generated conditions, depicted in Figure 10, were used to produce a new screening design that covers most of the design space. The resulting goodness-of-fit test results are reported in Table 15, and the χ^2^ values reveal an interesting outcome.

**Figure 10:**
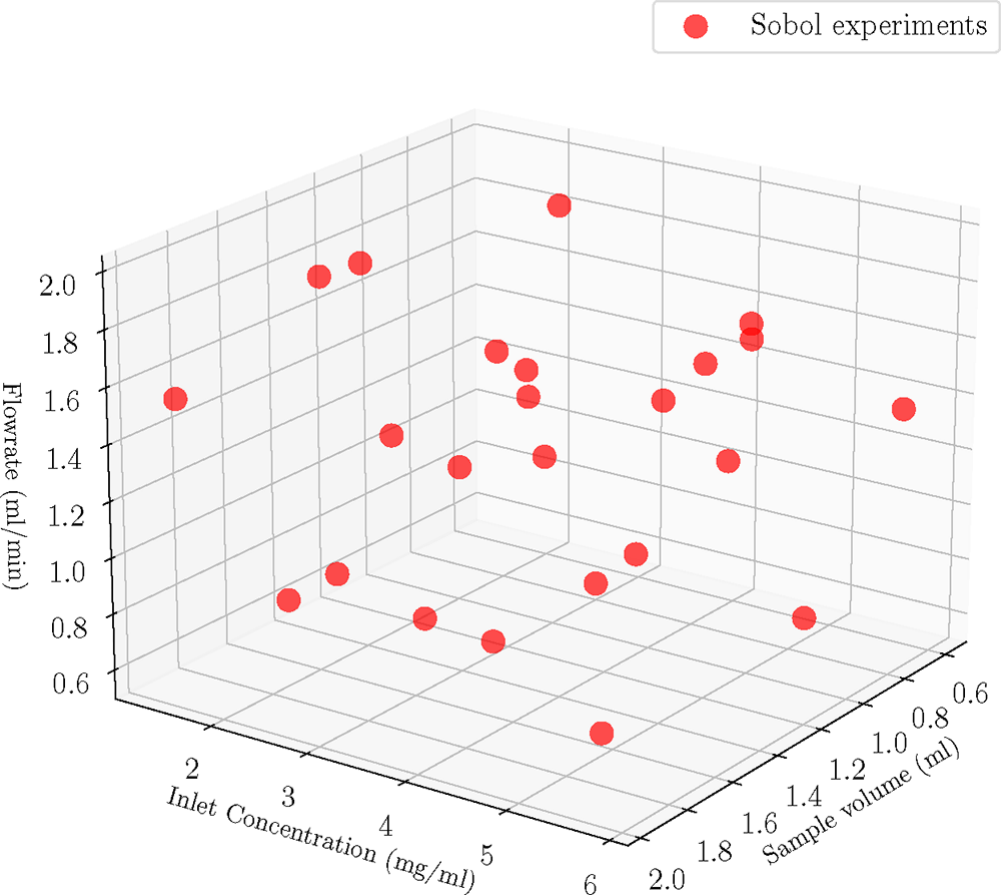
Case C: The 24 Sobol sampled experimental conditions used for validation.

**Table 15:** Case C: Goodness-of-Fit Test of the Proposed Models against 24 Quasi-Random Sobol Sampled Experiments

	**goodness-of-fit test**		
**model structure**	**χ^2^ (95%)**	**χ_*c*_^2^**	**outcome**
*a*_1_ *C*_*m*_ + *a*_2_ *C*_*m*_^2^ (eq 31)	1,600,000	2198	failed
*a*_1_ *C*_*m*_ + *a*_2_ *C*_*m*_^2^ + *a*_3_ *C*_*m*_^3^ (eq 32)	7365	2198	failed
*a*_1_ *C*_*m*_ + *a*_2_ *C*_*m*_^2^ + *a*_3_ *C*_*m*_^3^ + *a*_4_ tanh(*C*_*m*_)*C*_*m*_^3^ (eq 33)	15,475	2198	failed
*a*_1_ *C*_*m*_ + *a*_2_ *C*_*m*_^2^ + *a*_3_ *C*_*m*_^3^ + *a*_4_ *C*_*m*_^4^ (eq 34)	8537	2198	failed

The second-degree polynomial (eq 31) underfitted the experiments the most, as indicated by the χ^2^ values; an expected outcome owing to its simplest structure. In terms of the total χ^2^, the third-degree polynomial (eq 32) outperforms the rest of the models, with the fourth-degree polynomial performing slightly worse, and the hyperbolic tangent model (eq 33) coming third. However, according to Figure 11 which shows the ratio between the χ^2^ and χ_*c*_^2^ (in logarithmic scale) for each individual experiment, the fourth-degree polynomial (Figure 11) generalizes better for most of the experiments in relation with the third-degree polynomial (Figure 11). Also, the hyperbolic tangent modified polynomial (eq 33) performs well only in few of the experiments (Figure 11), while the second-degree polynomial (eq 31) is mostly inaccurate (Figure 11).

**Figure 11:**
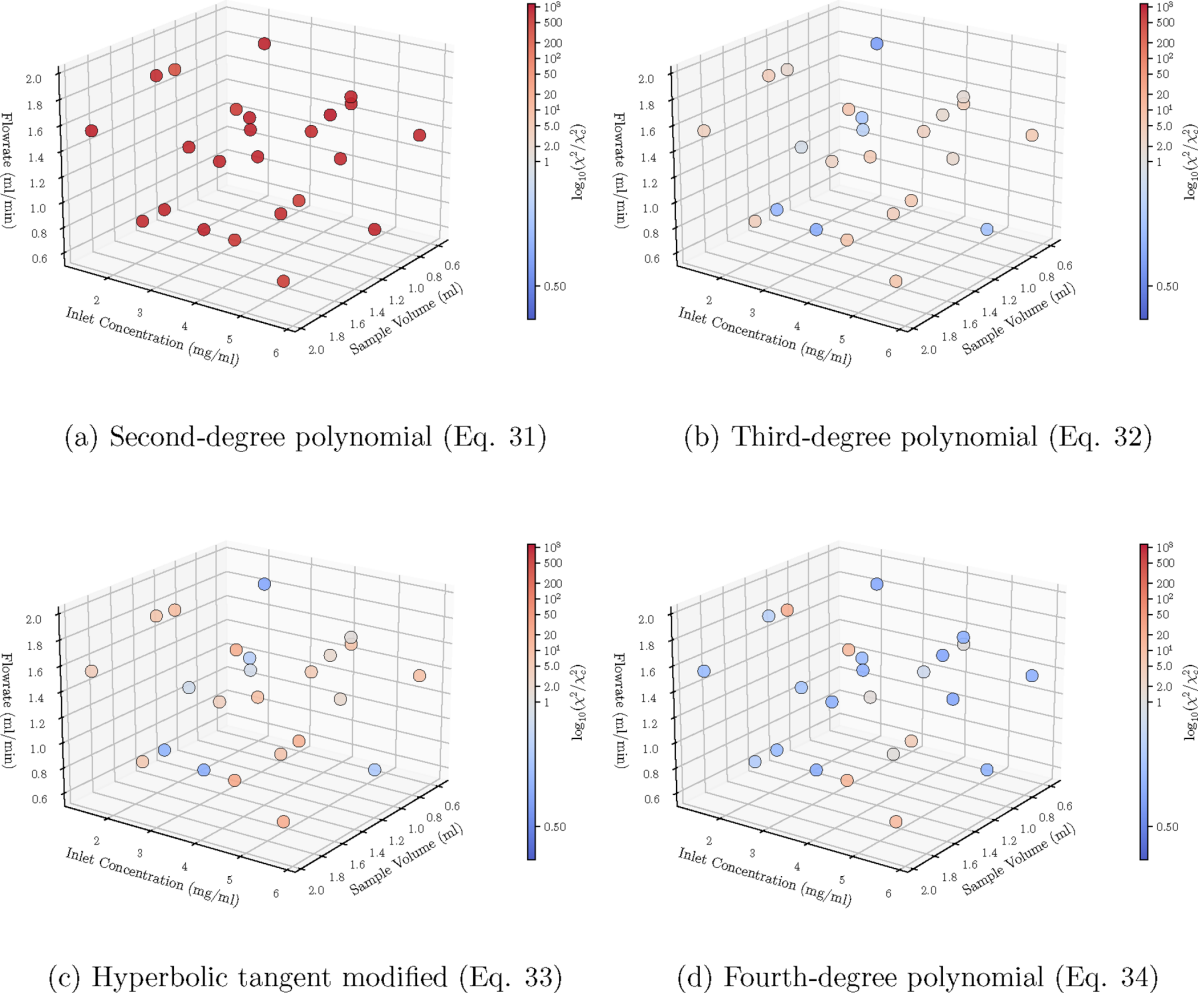
Case C: Schematic depiction of the logarithmic ratio between χ^2^ and χ_*c*_^2^ across the 24 Sobol sequence experiments.

These final results were based on 24 in-silico Sobol experiments, while in a real-world investigation, we would aim to avoid having to perform that many experiments. Hence, we would normally terminate the diagnostic methodology with the model structure that passed the goodness-of-fit test, for Case C that is the fourth-degree polynomial. Note that in a parameter estimation exercise, more complex model structures are expected to fit the experimental data best, as demonstrated from the χ^2^ values in Table 12. However, such models are not necessarily statistically more adequate across the overall design space, as demonstrated by the χ^2^ values in Table 15, likely due to limitations arising from the original sampling of the experimental data (used for parameter estimation) and potential loss in generalization. (To mitigate the loss of generalization, the modeler could potentially design a few additional, optimally designed, experiments via an exploratory design criterion such as G-Optimal.^47^)

It is crucial to acknowledge that all the evaluated models for Case C failed the goodness-of-fit test against the 24 Sobol generated experiments, albeit three of them are not far from the associated critical value. But, as seen from Table 12, the fourth-degree polynomial passed this test for the initial DoE considered there, which is all one would have in a real application. We therefore need to consider whether any of the models are potentially still usable, despite failing the χ^2^ test. From Figure 12, we can see that, while the second-degree polynomial does not produce chromatograms of high fidelity to the experiments, the rest of the models perform very well if just considering the chromatograms, and sufficiently well for most model applications. The high χ^2^ values stem from the models predicting values that are slightly higher or lower than it is accounted for in the χ^2^ distribution. These simulated points, although strictly speaking “out-of-bounds”, can still accurately capture the chromatogram trend for all the experiments. Hence, the χ^2^ test might not be the best metric for chromatogram curve-fitting, as it can clearly overpenalize. Practically, we could also accept the third-degree polynomial as the final modified model, although above we opted for a more conservative approach adhering to the original procedure proposed for kinetic models by Quaglio et al.^28^ In future work, alternative goodness-of-fit metrics, such as the ones proposed in the work of Heymann et al.,^48^ might be considered.

**Figure 12:**
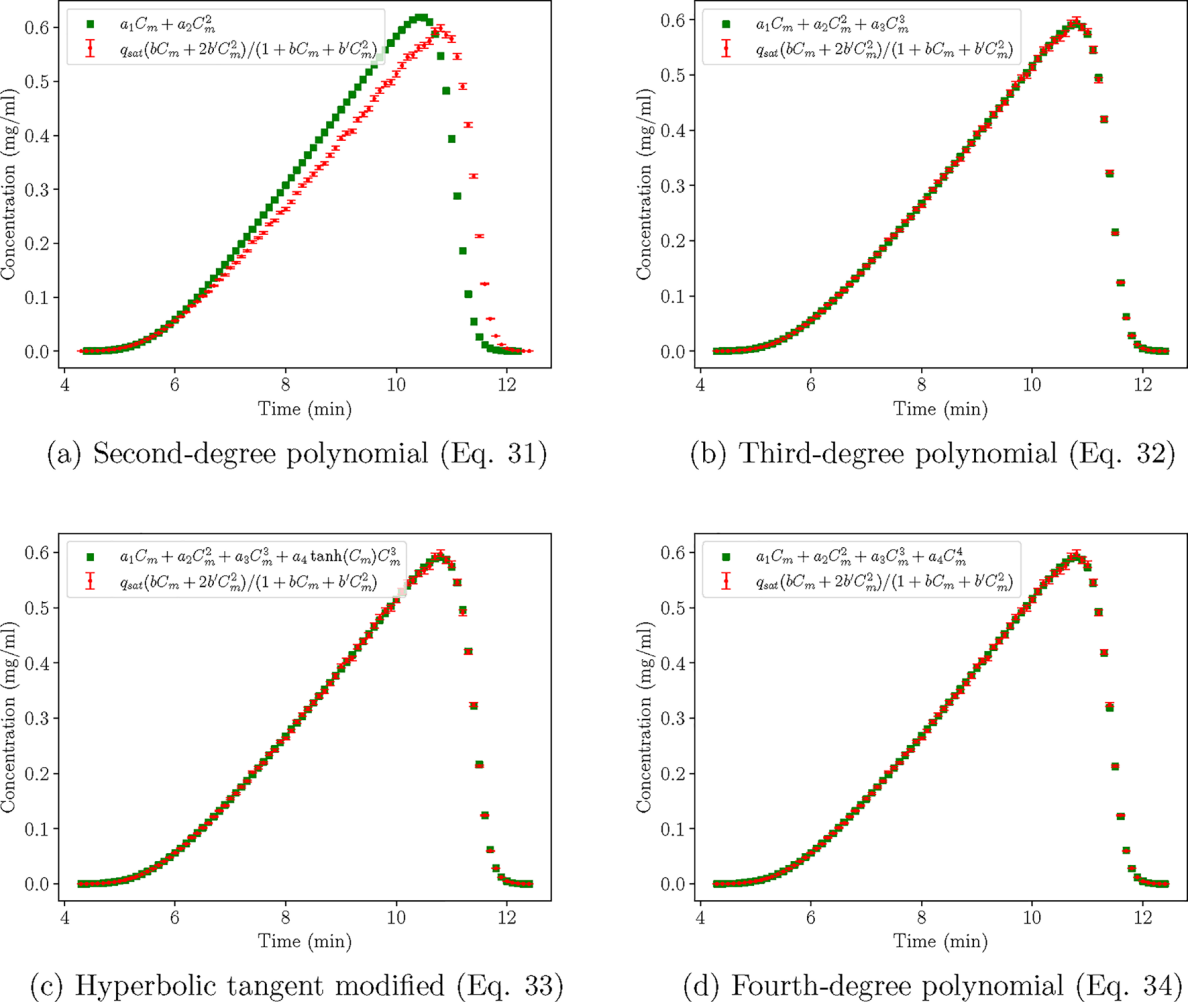
Case C: Comparison of the chromatograms produced by the different models (*V* = 0.96875 mL, *C*_in_^★^ = 1.9375 mg/mL, *Q* = 0.96875 mL/min).

## Conclusions

This work has proposed a diagnostic procedure for identifying suitable isotherm models in the development of liquid chromatography models. The procedure is based on a maximum likelihood inference framework, combined with a goodness-of-fit test and a Lagrange multiplier test to evaluate model performance. In particular, the Lagrange multiplier test is used to detect parameters that are not constant but instead vary as functions of the system’s state variables.

A key contribution of this work is the application of the procedure to chromatography, demonstrating its potential to be extended toward automated identification of complex isotherm models, particularly through the use of flexible representations such as polynomials, or by implementing the method into supervised search-based model building algorithms. This capability is highly relevant to applications in pharmaceutical purification, bioprocessing as well as chemical kinetics, where accurate and interpretable modeling is essential.

We demonstrated the capabilities of the procedure by exploring three case studies. In Cases A and B, the initial isotherm models were modified according to the diagnostic procedure, which gave rise to more complex but more accurate models that passed the goodness-of-fit test. In Case C, no known isotherm model could fit the experimental data set adequately according to the tests used. It was shown how the procedure could nevertheless be used to iteratively modify a quadratic polynomial until a model structure was reached that passed the goodness-of-fit test.

Although the proposed diagnostic procedure does not consider any intuition in detecting faulty parameters, it still relies on the expertise of the modeler in proposing appropriate constitutive equations. Future work should focus on methods that contribute to more informed decision making in parameter substitution, in order to limit potential bias in the model selection procedure, as well as exploring different methods for evaluating the goodness-of-fit. Additionally, applying the methodology to a real-world case study is of significant value to evaluate the capabilities and limitations of the methodology.

## Supplementary Material



## Notes

Full derivation of the Taylor expansion, derivation of the radius of convergence (PDF)
