# Supplementary material for: A Diagnostic Procedure for Identifying Isotherm Models in Liquid Chromatography
Source: Ind Eng Chem Res. 2026 Jan 6;65(2):1277–92. doi: 10.1021/acs.iecr.5c03704 (PMC12828779; doi:10.1021/acs.iecr.5c03704)
Supplement: Supplementary file 1 [file ie5c03704_si_001.pdf]

Supporting Information: A diagnostic procedure for identifying isotherm models in liquid chromatography

Konstantinos Katsoulas, Federico Galvanin, Luca Mazzei, and Eva Sorensen

Department of Chemical Engineering, Sargent Centre for Process Systems Engineering, University College London, Torrington Place, London, WC1E 7JE, United Kingdom

December 2025

Taylor Expansion

This document of supporting information goes through the derivation of the Taylor expansion of the quadratic isotherm used in the main text, whilst it also obtains the radius of convergence of the said expansion. We expand the quadratic isotherm of Eq. 28 around $C_m = 0$. The Taylor expansion reads:

$$q(C_m) = q(0) + q'(0)\,C_m + \frac{q''(0)}{2!}C_m^2 + \frac{q'''(0)}{3!}C_m^3 + \frac{q''''(0)}{4!}C_m^4 + \dots \quad (1)$$

For the sake of simplicity in our mathematical passages, we re-write the quadratic isotherm, a rational function, in terms of z:

$$q(z) = q_{sat}\frac{bz + 2b'z^2}{1 + bz + b'z^2} = \frac{P(z)}{T(z)} \quad (2)$$

with:

$$P(z) \equiv q_{sat}\left(bz + 2b'z^2\right)\ ;\ T(z) \equiv 1 + bz + b'z^2 \quad (3)$$

We can expand $\frac{1}{T(z)}$ analogously to the Taylor expansion of $\frac{1}{1+x}$. For instance, the Taylor series of $\frac{1}{1+x}$ around $x = 0$ is:

$$\frac{1}{1+x} = 1 - x + x^2 - x^3 + x^4 - \dots \ \text{ for } \ |x| < 1 \quad (4)$$

Similarly, we can expand $\frac{1}{1+bz+b'z^2}$ by setting $x = bz + b'z^2$, whence we obtain:

$$\frac{1}{1 + bz + b'z^2} = 1 - bz - b'z^2 + b^2z^2 + 2bb'z^3 + b'^2z^4 - b^3z^3 - 3b^2b'z^4 + b^4z^4 + \dots \quad (5)$$

If we multiply the expanded terms of Eq. 5 with the numerator, $P(z)$, we get the following fourth-degree expansion for the quadratic isotherm:

$$q = q_{sat}\left[bz + (-b^2 + 2b')z^2 + (-3bb' + b^3)z^3 + (4b^2b' - 2b'^2)z^4 + \mathcal{O}(z^5)\right] \quad (6)$$

For the $\frac{1}{1+x}$ function, the convergence radius was $|x| < 1$; thus, we similarly have to estimate the convergence radius of the expansion in Eq. 6. The Cauchy-Hadamard theorem states [1, 2] that *given a power series* $\sum_{n=0}^{\infty} a_n z^n$, *there exists* $0 \leq R \leq \infty$ *such that*:

1. *If* $|z| < R$ *the series converges absolutely*
2. *If* $|z| > R$ *the series diverge.*

The radius of convergence R centred at zero is equal to the distance to the nearest singularity (pole) in the complex plane. To find the closest pole, we need to find the roots of the denominator of the function. Thus:

$$1 + bz + b'z^2 = 0 \quad (7)$$

By solving the above, we can obtain the roots of z:

$$z = \frac{-b \pm \sqrt{b^2 - 4b'}}{2b'} \quad (8)$$

Substituting the parameter values, we obtain:

$$z = \frac{-0.04 \pm \sqrt{-0.0784}}{0.04} \Rightarrow z \approx -1 \pm 7i \quad (9)$$

The magnitude of the complex roots give the radius of convergence, R:

$$|R\,| \approx 7 \quad (10)$$

References

[1] Elias M. Stein. *Complex analysis.* Princeton lectures in analysis. Princeton University Press, Princeton, N.J., 2003.

[2] E. T Whittaker, G. N Watson, and Victor H Moll. *A Course of Modern Analysis.* Cambridge University Press, Cambridge, 5th edition. edition, 2021.
